# Positron emission tomography detection of human endothelial cell and fibroblast monolayers: effect of pretreament and cell density on ^18^FDG uptake

**DOI:** 10.1186/2045-824X-4-5

**Published:** 2012-03-20

**Authors:** Julie A Chouinard, Jacques A Rousseau, Jean-François Beaudoin, Patrick Vermette, Roger Lecomte

**Affiliations:** 1 Sherbrooke Molecular Imaging Centre, Department of Nuclear Medicine and Radiobiology, Université de Sherbrooke, 3001, 12ième Avenue Nord, Sherbrooke, Québec J1H 5N4, Canada; 2 Laboratoire de bio-ingénierie et de biophysique de l'Université de Sherbrooke, Department of Chemical and Biotechnological Engineering, Université de Sherbrooke, 2500, boul. de l'Université, Sherbrooke, Québec J1K 2R1, Canada; 3 Research Centre on Aging, Université de Sherbrooke, 1036, rue Belvédère Sud, Sherbrooke, Québec J1H 4C4, Canada

**Keywords:** FDG, Positron Emission Tomography (PET), HUVEC, Endothelial Cells, Fibroblasts, Tissue Engineering

## Abstract

**Background:**

The non-destructive assessment and characterization of tridimensional (3D) cell and tissue constructs in bioreactors represents a challenge in tissue engineering. Medical imaging modalities, which can provide information on the structure and function of internal organs and tissues in living organisms, have the potential of allowing repetitive monitoring of these 3D cultures *in vitro*. Positron emission tomography (PET) is the most sensitive non-invasive imaging modality, capable of measuring picomolar amounts of radiolabeled molecules. However, since PET imaging protocols have been designed almost exclusively for *in vivo *investigations, suitable methods must be devised to enable imaging of cells or tissue substitutes. As a prior step to imaging 3D cultures, cell radiotracer uptake conditions must first be optimized.

**Methods:**

In this study, human umbilical vein endothelial cells (HUVEC) and human fibroblasts were cultured at different densities and PET was used to non-destructively monitor their glycolytic activity by measuring ^18^F-fluorodeoxyglucose (^18^FDG) uptake. Various cell preconditioning protocols were investigated by adjusting the following parameters to optimize ^18^FDG uptake: glucose starvation, insulin stimulation, glucose concentration, ^18^FDG incubation time, cell density and radiotracer efflux prevention.

**Results:**

The conditions yielding optimal ^18^FDG uptake, and hence best detection sensitivity by PET, were as follows: 2-hour cell preconditioning by glucose deprivation with 1-hour insulin stimulation, followed by 1-hour ^18^FDG incubation and 15-minute stabilization in standard culture medium, prior to rinsing and PET scanning.

**Conclusions:**

A step-wise dependence of ^18^FDG uptake on glucose concentration was found, but a linear correlation between PET signal and cell density was observed. Detection thresholds of 36 ± 7 and 21 ± 4 cells were estimated for endothelial cells and fibroblasts, respectively.

## Introduction

The growth of tissue substitutes is a dynamic process, requiring close monitoring of cell viability and function over time. Unfortunately, most of the commonly available cell assays (e.g., histology, immunofluorescence or immunocytochemistry) are time consuming and require sacrificing the culture. Although these techniques provide important information on cell phenotype and function, they are often only representatives of specific time points and selected samples within the culture. It remains difficult to trace the evolution of the cell or tissue cultures over time. Our inability to obtain continuous direct information on culture conditions and cells state in thick (few mm to cm range) 3D culture chambers represents a major weakness in understanding bioreactors performance. The main challenge nowadays is to find suitable real-time, non-invasive and, most importantly, non-destructive characterization methods to monitor these large non-transparent cell and tissue cultures. As reviewed by Dubois et al. [1], imaging techniques borrowed and adapted from the biomedical field can be used to monitor the dynamic tissue growth process without interference. While imaging modalities such as X-ray computed tomography (CT) and magnetic resonance imaging (MRI) can provide subtle morphological details, functional imaging using picomolar amounts of radiolabeled molecules can be used to obtain sensitive information on the underlying biological and biochemical processes non-destructively. Positron Emission Tomography (PET), in particular, offers great potential to monitor cell metabolism, proliferation, angiogenesis, perfusion, hypoxia or apoptosis using a range of specific radiotracers without hindering normal tissue development. [^18^F]-fluorodeoxyglucose (^18^FDG), a glucose analog labeled with the positron-emitting radioisotope fluorine-18 that can be used as a cell glycolysis marker, is the most commonly used PET tracer. ^18^FDG uptake can provide valuable information on cell viability, proliferation and initial tissue perfusion in bioreactors [2-4]. In humans, vascular ^18^FDG uptake is usually undetectable except in some cases of vasculitis or atherosclerosis [5]. Knowing the importance of microvasculature in tissue engineering, we focussed our efforts on increasing ^18^FDG uptake by fibroblasts and endothelial cells [6]. To improve the PET signal from the target tissue, a proper imaging protocol had to be devised and the uptake parameters of the radiotracer by cells in the culture optimized. As the tissue culture in bioreactors is dependent on several environmental parameters, a screening method to assess the best radiotracer uptake conditions for cell types of interest was first developed.

This study describes a fast *in vitro *screening method allowing many parameters with potential impact on cell ^18^FDG uptake to be evaluated concurrently. A 2-step method was designed to quantitatively measure the radiotracer PET signal by imaging living cell monolayers under various culture conditions, allowing the cell ^18^FDG uptake parameters to be optimized. The proposed steps are 1) a factorial design and 2) a detailed screening for each major acting parameter found in step 1.

## Materials and methods

Human umbilical vein endothelial cells (HUVEC, C-12203) and human dermal fibroblasts (C-12350) were purchased from PromoCell (Heidelberg, Germany). Fetal bovine serum (FBS, F-1051), Medium 199 (M199, M5017), endothelial cell growth supplement (ECGS, E2759), heparin (H1027), gelatin type B (G9391) and Hoechst 33342 (14533) were all obtained from Sigma-Aldrich (Oakville, ON, Canada). Phosphate Buffered Saline (BP665-1), and disposable plastic wares came from Fisher Scientific (Whitby, ON, Canada). Trypsin-EDTA (25200-056) and antibiotics (penicillin G/streptomycin sulphate (15140-122)) were obtained from Invitrogen (Burlington, ON, Canada). 5% dextrose water solution (JB0062) came from Baxter (Mississauga, ON, Canada), glucose-free DMEM (319-061-CL) from Wisent (St-Bruno, QC, Canada) and human Novolin Toronto insulin (DIN 02024233) from Novo Nordisk (Mississauga, ON, Canada). Nunc's Lab-Tek II 8 chamber slides (62407-296) were purchased from VWR (Mississauga, ON, Canada).

### Cell culture

HUVEC and fibroblasts were cultured in M199 supplemented with 10% FBS, heparin (90 μg/mL), L-glutamine (2 mM), penicillin G (50 U/mL) and streptomycin sulphate (50 μg/mL). ECGS (20 μg/mL) was added to HUVEC culture media. Cells were kept in an incubator (5% CO_2 _in humid atmosphere). HUVEC at passage 3 or 4 were used to avoid senescence [7]. Cell seeding was always performed on surfaces coated with gelatin (gelatin solution concentration of 100 μg/mL) to promote cell adhesion and limit batch-to-batch variation towards cell attachment.

### Radiochemistry

Fluorine-18 was prepared by the ^18^O(p, n)^18^F reaction on ^18^O enriched water as target material using a TR-19 cyclotron (Advanced Cyclotron Systems, Vancouver, BC, Canada). For the synthesis of [^18^F]fluorodeoxyglucose (^18^FDG), an established procedure was used [8].

### Step 1 - Factorial design to identify variables affecting ^18^FDG uptake by cells

**Figure 1:**
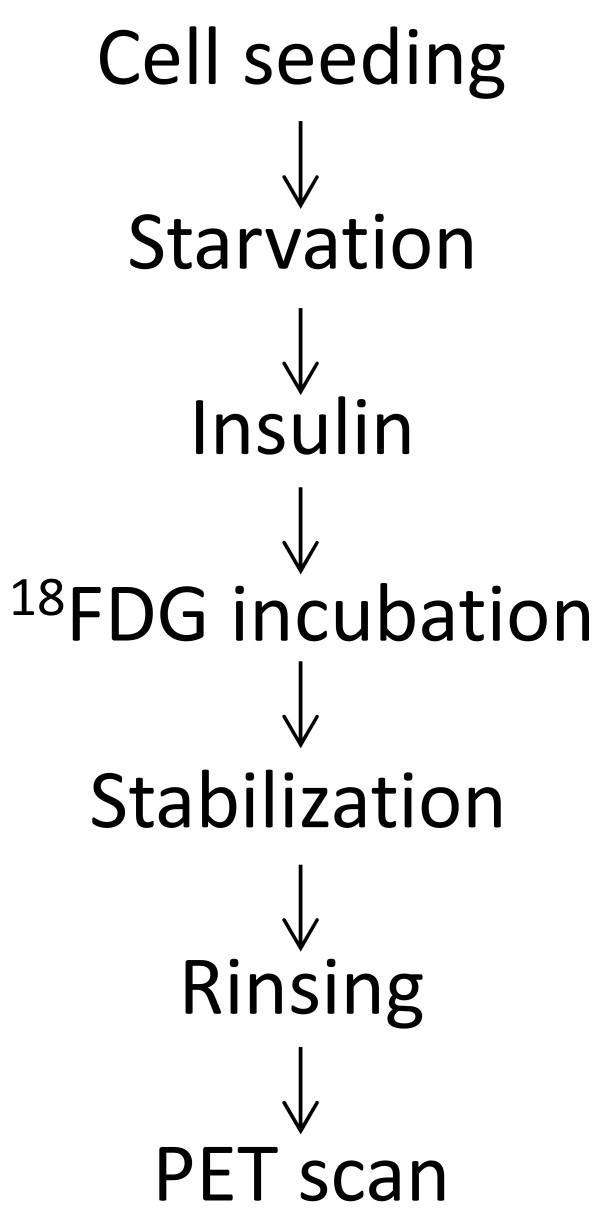
**Experimental timeline for the factorial design**.

To optimize cell signals for PET detection, a factorial design was employed to study the effects of variables among ^18^FDG incubation time, cell density, insulin exposure and concentration, glucose concentration and culture stabilization time on ^18^FDG uptake by cell monolayers. A non-replicated one block 2 k-2 factorial design with 3 center points was used for the ^18^FDG cell uptake experiments. This method in conjunction with ANOVA is well adapted for the identification of the conditions having the most impact on cell uptake of ^18^FDG. Upper and lower thresholds were defined for each variable investigated in this study, based on our personal experience and the limitations imposed by ^18^FDG half-life. The variables studied and their corresponding thresholds are presented in Table 1 and parameters were classified in order of influence. The selected limits combined with the factorial design of experiments including center points and 2 rinsing controls yielded a total of 16 experiments. Design of experiments and statistical analyses of the results were done using the Stat-Ease software (Stat-Ease Inc., Minneapolis, MN), using ANOVA. A *P*-value equal or smaller than 0.05 was considered statistically significant. The experimental steps and the followed order are presented in Figure 1.

**Table 1:** Thresholds of the experimental design for all cell types in order of influence

Variables	Lower threshold	Upper threshold	^18^FDG uptake gain	*P*-value
Starvation time	0 h	3 h	43.9%	< 0.0001*

Cell density	10 000 cells/mL	50 000 cells/mL	20.3%	0.0002*

^18^FDG incubation time	1 h	2 h	5.8%	0.0062*

Insulin concentration	0.0 nmol/mL	6.0 nmol/mL	1.4%	0.1016

Insulin incubation time	0 h	1 h	1.0%	0.1475

* *P*-values of less than 0.05 indicate model parameters are significant

### Step 2 - Cell preconditioning to increase ^18^FDG uptake

**Figure 2:**
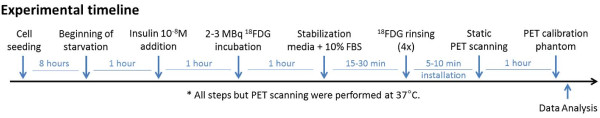
**Experimental timeline for step 2**.

In light of the factorial design results, a new experimental design and timelime was determined. The new protocol is summarized in Figure 2. The starvation period was shortened to 2 hours and the 1-hour ^18^FDG incubation was preferred since the gain for the extra hour was not deemed sufficient considering the loss caused by the 109.8 minutes half-life of the ^18 ^F radiotracer. The used insulin concentration was also lowered down to a physiological level. The most important parameter affecting cell ^18^FDG uptake (glucose starvation) was further investigated to find which optimal glucose concentration should be used without affecting the samples. Once found, the influence of cell density on the measured signal was tested.

#### Samples preparation

**Figure 3:**
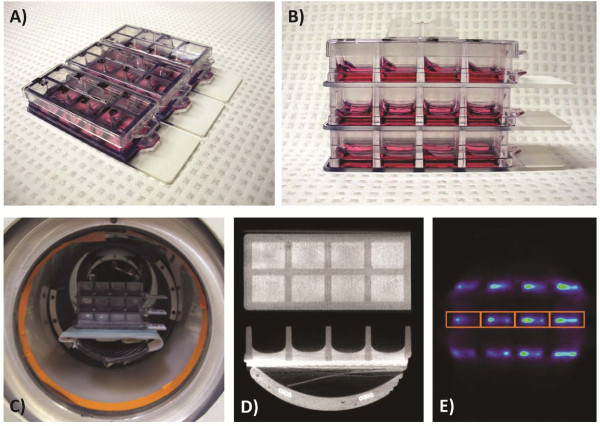
**A) Cells seeded in chamber slides, B) Stacked chamber slides for PET scanning, C) Aligning stack in PET axial and trans-axial field of view (FOV), D) CT images used to draw regions of interest (ROI), E) PET image with an example of projected CT-defined ROI for PET data analysis**.

After seeding, cells were given 8 hours to adhere and spread in the chamber slides (Figure 3A). This period was enough to avoid energetic demand caused by the cell attachment procedure and short enough to prevent density variation due to proliferation before starting any treatment.

#### Starvation

Glucose starvation media consisted in glucose-free DMEM with 1% FBS supplemented with a 5% dextrose solution to set the desired glucose concentrations (0 to 7 mM). Each well was rinsed thrice with starvation media and left to incubate at 37°C with 5% CO_2 _under humid atmosphere. The incubation media volume represented 20% of the overall volume capacity of the well. Since this parameter could deeply affect cells, we tested the effect of glucose starvation on cell morphology and cell number to validate a safe working range. Cells were incubated for 2 hours in supplemented DMEM with different glucose concentrations (n ≥ 3) ranging from 0 to 7.0 mM with increments of 0.5 mM. Cell morphology was assessed before and after each glucose starvation treatment. To count cells, samples were incubated 30 min in 1/1000 Hoechst 33342 and were then scanned with an iCys imaging cytometer (Compucyte, Westwood, MA) using a violet diode excitation laser (405 nm) with blue detector (465/20 nm) for DNA staining and scatter detector for visualization of cytoplasm. Scanning was performed at a 1 μm × 0.5 μm pixel size resolution. Cell selection was done using DNA staining taking into account cell DNA content, allowing to score correctly for cell clumps and to reject small fragments containing DNA.

#### Insulin

A 10^-8 ^M physiological insulin concentration was added to every well 1 hour after treatment onset and 1 hour before ^18^FDG injection since its effects take 30-45 minutes to occur before lasting a few hours. 1 mole of insulin corresponds to 166.8 × 10^6 ^IU or 1.0 IU = 6.0 nmol [9].

#### ^18^FDG incubation

1 hour post-insulin, the radiotracer was supplemented in excess using 2-3 MBq (15 μL) of ^18^FDG in every well.

#### Stabilization

After a 1-hour incubation with ^18^FDG, the wells were filled up to 50% of their volume capacity using M199 containing 10% FBS for incubation periods ranging from 15 to 60 minutes to evaluate the ^18^FDG efflux.

#### Rinsing

After stabilization, wells were all carefully rinsed 4 times with the corresponding fresh starvation solution to remove free ^18^FDG and the fourth rinse was counted to check for the presence of residual radioactivity before scanning. Every last rinse had to be ^18^FDG-free to ascertain that the PET signal only originated from cell uptake. Each chamber slide also had one cell-free well that was treated like the others (^18^FDG and rinses) and served as a background reference.

### Cell culture imaging

Positron emission tomography (PET) imaging was used to monitor the cell ^18^FDG uptake together with X-ray computed tomography (CT) imaging to define the morphology of the chamber slides. A Triumph™ PET/CT dual modality imaging platform (Gamma Medica, Inc., Northridge, CA, USA) was used, which consisted of a LabPET™ avalanche photodiode-based digital PET scanner with a 7.5 cm axial field-of-view [10] capable of achieving a transaxial spatial resolution of 1.2 mm and a detection efficiency of 2.1% with an energy window setting of 250-650 keV.

Chamber slides were mounted in stacks (Figure 3B) and centered in the scanner transaxial and axial field of view (FOV) (Figure 3C). One-hour static PET data acquisitions with axial double sampling motion to improve resolution were performed for every cell imaging session, followed by 5-minute static imaging of a phantom containing a known amount of ^18^FDG at the end of the imaging period to allow for the conversion of detected events (in CPS/Pixel) into MBq of retained activity in cells. The calibration phantom consisted of cell-free chamber slides with two known ^18^FDG concentrations in volumes of 200 μL pipetted in non-consecutive chambers. The scanner efficiency was further normalized within a day of the measurement with a ^68^Ge 18.5 MBq rotating line source (PET-78/0.5, Sanders Medical, Knoxville, TN) for at least 4.5 hours to ensure reproducibility between measurements, which were taken up to several weeks apart. PET images were reconstructed on a 0.25 mm × 0.25 mm × 1.1175 mm grid using 20 iterations of a 2D maximum-likelihood expectation maximization (MLEM) algorithm implementing position-dependent detector response [11]. Corrections for individual detector efficiency and random coincidences were applied, but attenuation and scatter corrections were omitted since they were unnecessary given that all measurements were taken with the same stacked thin chamber slides geometry. CT acquisition was performed in fly mode with 512 projections in 2.13 min at 60 KVP and 220 μA. The single frame was reconstructed in 0.17 × 0.17 × 0.17 mm^3 ^voxels. The Amide freeware (sourceforge.net version 0.9.2) was used to analyze the radiotracer concentration in the reconstructed images, using the CT scan image of the culture slide to draw 8 equal regions of interest (ROI) over each chamber (Figure 3D). These ROI were then projected on the corresponding PET images for data analysis (Figure 3E).

## Results

### Factorial design

The results of the factorial design experiments are summarized in Table 1. Design showed that all, but insulin concentration and incubation time had a *P*-value < 0.05. The 3-hour glucose starvation period before exposing cells to ^18^FDG was the most significant factor, which alone represented 44% of the total radiotracer uptake enhancement. Cell density was the second most important factor with a 20% impact.

Different stabilization periods were evaluated to facilitate cell ^18^FDG trapping and avoid efflux. These processes are dependent on cell hexokinase activity [12], the rate-determining step for metabolic trapping [13,14] and ^18^FDG-6-phosphate dephosphorylation by glucose 6-phosphatase [15,16]. Indeed, hexokinase had been shown to reach a maximum activity starting at 5 mM of glucose and over [17]. To maximise ^18^FDG confining and minimise efflux, once the radiotracer incubation time was elapsed, we added 5.5 mM glucose containing 10% FBS to each well for periods of 0, 15, 30 or 45 minutes before rinsing. We found that a stabilization time between 15 to 30 minutes gave a stable signal and this period was considered optimal for both cell types.

### Effect of glucose starvation on cell morphology

**Figure 4:**
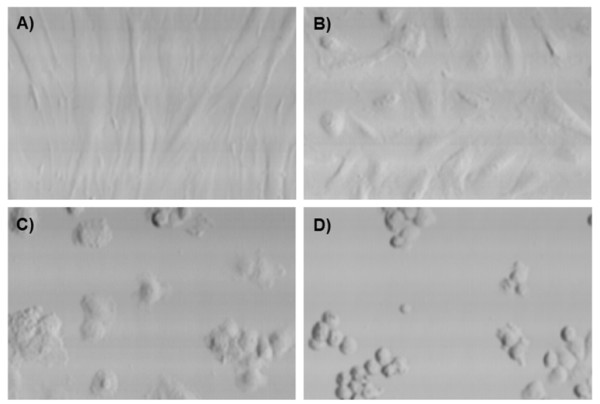
**Laser scanning cytometry images of fibroblasts (A) and HUVEC incubated 2 hours in DMEM containing 2, 0.5 and 0 mM glucose (B to D respectively)**. A is representative of all fibroblast samples. B is representative of all glucose concentrations above 2 mM.

When using PBS as a glucose-free buffer at 37°C, all cells, especially HUVEC, became spherical and detached from the surface, which is consistent with previous observations [18]. A treatment that changes samples morphology would not be appropriate for bioreactor cultures. This problem was solved using glucose-free DMEM instead. Fibroblasts did not undergo morphological changes under any conditions with this starvation medium (*n *= 3) (Figure 4A). HUVEC (Figure 4B), on the other hand, showed morphological changes with glucose concentration lower than 1.5 mM (Figure 4C) and were completely round after 2 hours in 0.5 mM (Figure 4D). Most cells shown in Figure 4D did not recover from glucose starvation after 48 hours in commercial 5.5 mM glucose containing M199.

### Effect of glucose starvation on ^18^FDG uptake

**Figure 5:**
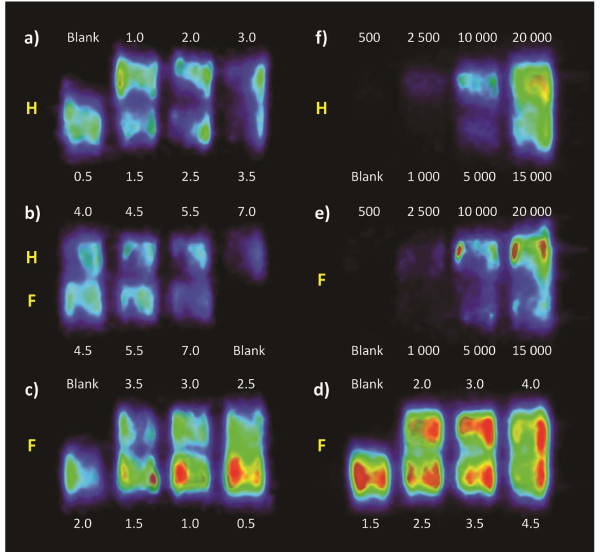
**^18^FDG/PET images of HUVEC (H) and fibroblasts (F) pretreated with media containing glucose concentrations from 0.5 to 7.0 mM (a to c)**. Panel (d) shows a closer look at the plateau found with fibroblasts between glucose concentrations of 1.5 and 4.5 mM. Note that the color scale has been stretched in (d) to better appreciate the subtle differences between relatively similar samples. Numbers above and below the PET images represent the media glucose concentration in mM. Panels *e *and *f *show PET images of fibroblast and HUVEC monolayers at different cell densities treated with 3 mM glucose. Numbers above and below these images indicate the numbers of cells at seeding (8 hours prior to scanning).

**Figure 6:**
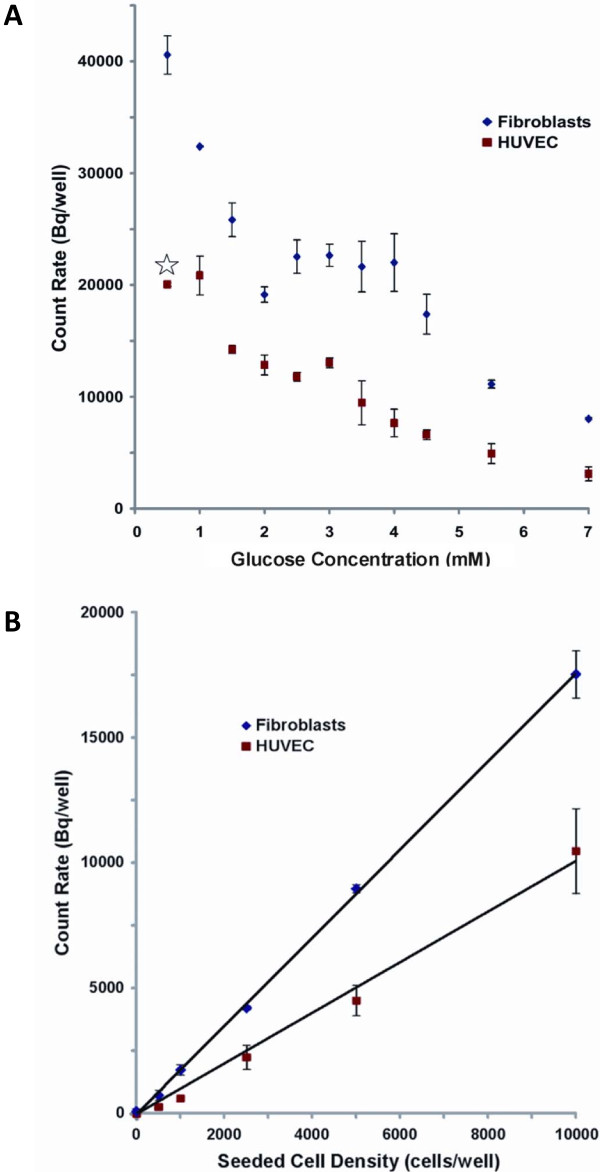
**Quantitative PET data analysis (*n *≥ 3) as a function of glucose concentration during the 2-hour starvation (A) and cell density at seeding (B)**. The star in (A) indicates that cell morphology was deeply affected (see Figure 4C).

HUVEC and fibroblast monolayers were each treated with media containing different glucose concentrations and subsequently scanned by PET to assess their ^18 ^FDG uptake, as shown in Figure 5a, b, and 5c. A plateau was observed in scans of fibroblasts between 2.5 and 4.0 mM of glucose (Figure 5d), so further investigation were carried out in that range to confirm these observations. Quantitative PET data obtained by ROI analysis, after correction for radioactive decay, are reported in Figure 6A. For fibroblast monolayers, ^18^FDG signal decreases steeply in a linear fashion (R^2 ^= 0.9972) from 0.5 to 2 mM of glucose, then slightly rebounds to a plateau between 2.5 and 4 mM, to fall again most probably because the glucose concentration exceeded cell needs and entered in competition with ^18^FDG. For HUVEC, excluding the result for 0.5 mM glucose treatment that deeply affected cell morphology (Figure 6A, star), the ^18^FDG signal drops steeply between 1.0 and 1.5 mM, then levels off between 1.5 and 3 mM, before steadily decreasing for higher glucose concentrations.

### Effect of cell density on PET signal

A linear correlation was found between ^18^FDG signal and the number of cells. Assuming that a signal-to-noise ratio equal or larger than 3 is significant, detection thresholds of 36 ± 7 cells for HUVEC and 21 ± 4 cells for fibroblasts were extrapolated from the linear correlations used to fit data in Figure 6B. The difference of slope between HUVEC and fibroblasts in this experiment reflects the higher ^18^FDG uptake by the latter, in agreement with results obtained in Figure 6A.

## Discussion

Glucose deprivation is known to gradually increase total cellular transporter proteins [19,20] and is also associated with decreased protein turnover in mammalian fibroblasts [19]. Starved mammalian cells under low glucose concentration conditions undergo a p53 dependent G1 phase arrest that is quickly reversible upon glucose restoration [21]. Fibroblasts have been showed to have a 24-hour survival capacity starting from 0.1 mM of glucose [21], which is in agreement with our results. Highest ^18^FDG uptakes were always obtained under a 2 mM glucose concentration, but such a low glucose concentration affected HUVEC morphology (Figure 4C-D). The spheroid HUVEC appearance observed under 1.5 mM glucose concentration can be due to cell-cell detachment caused by F-actin conversion in G-actin rather than being a consequence of cell death induction [22]. However, not every preconditioning treatment susceptible to influence cell morphology can be considered for imaging cell or tissue substitutes.

When working with HUVEC, the optimal ^18^FDG uptake, while still maintaining cell integrity, was achieved using 3 mM glucose containing DMEM. The 3 mM glucose concentration also corresponded to the uptake plateau for fibroblasts. Hence, this glucose concentration was used for the cell density gradient experiments shown in Figure 5e and 5f and analysed in Figure 6B. Nearly all cell culture media contain a glucose concentration of at least 5.5 mM, which can be considered as a tremendous source of carbohydrate able to last for days [23]. Most cells do not need that much glucose and quickly become saturated, making them produce and excrete lactate [23]. Lowering the glucose level to 3 mM for 2 hours increased ^18^FDG uptake to approximately twice that found when using 5.5 mM media for both HUVEC and fibroblasts. Endothelial cells were shown to present different glucose metabolism and insulin responsiveness according to their organ of origin, so caution should be exercised when applying these results to other cell types [24].

The FBS supplemented M199 medium used for the cell culture contained 4 mM glucose, but since it was used at a concentration of 1% in the cell starvation media, this amount of added glucose was considered as non significant. Hiraki et al. [25] reported that glucose transport is also regulated by calf serum growth factors in a concentration-dependent manner. Considering that serum induced a first rise of sugar uptake within 10 minutes and a second at approximately 1 hour due to the activation of glucose transporter gene expression [25], it might be possible to gain extra signal by using 15% FBS containing DMEM before adjusting the glucose level to 3 mM.

Over-expression of insulin-receptors in HUVEC showed the presence of a functional insulin pathway [26]. A small increase in ^18^FDG signal had been noticed in our data when using 10^-8 ^M insulin, but this effect turned out not to be statistically significant. Insulin action might not be a major actor in our system, but anything that could bring some improvement is always welcome, so a 10^-8 ^M physiological concentration was preserved in our protocol, as suggested by the maximum insulin effect observed by Gerritsen et al. [13,27]. It must also be kept in mind for further studies that tissues such as heart, skeletal muscles and adipose tissues do present the insulin responsive glucose transporter GLUT4 [28-30], so even if our results with fibroblasts (known to have GLUT1, 3 and 4 [31]) turned out not to be significant, we strongly suggest that the insulin parameter always be tested.

Now that a protocol to maximize ^18^FDG cell uptake has been established, further studies are being planned to investigate additional parameters that are known to influence glucose uptake in cells, such as the presence of nitric oxide [12,24,26], growth factors [32], hypoxia [33], and proliferation.

More imaging studies will be needed to fully understand the importance of these factors in high cell density cultures, and PET imaging offers considerable potential to achieve this goal. Numerous key parameters must be dynamically monitored in real time in tissue cultures to optimize their development, which include morphology, viability, proliferation, metabolism, angiogenesis, perfusion, nutrient and oxygen consumption, hypoxia, apoptosis, and sometimes secretion of specific proteins. So far, only ^18^FDG, a cell glycolytic activity marker, has been investigated, but several other PET tracers are available, such as ^18^F - fluorothymidine (^18^FLT) and ^11 ^C-methionine to, respectively, monitor DNA and protein synthesis, ^18 ^F-fluoromisonidazole (^18 ^F-MISO) for imaging hypoxia [34], and ^18^F- or ^64^Cu-labeled annexin-V for measurement of apoptosis [35,36]. It would also be possible to adapt the proposed protocol for non-adherent (floating) cells by using microtubes for imaging instead of the square culture chambers. A centrifugation step would then be required prior to media removal for rinsing. Since radiotracers can sometimes bind to the microtube's wall, it may be advisable to transfer the suspension in a fresh tube after every rinse to avoid contamination from the container in the PET images.

Extension of the protocol used in this study to other cell types would be straightforward, provided that they have a similar growth rate and ^18^FDG delivery times. Due to the 109.8 minutes half-life of fluorine-18, the used experimental set-up with ^18^FDG could hardly be feasible under longer working conditions than the 12-h protocol used here. Obviously, other biological parameters could be monitored over extended observation periods with possibly longer incubation times using molecular probes labelled with longer half-life radiotracers, such as ^64^Cu (12.8 h), ^89^Zr (78 h) or ^124^I (4.18 d).

## Conclusions

In this study, we have investigated the ^18^FDG uptake of two human cell types of importance in many tissue engineering applications. Parameters influencing ^18^FDG uptake by HUVEC and human fibroblasts have been optimized by directly imaging living cell monolayers with PET using a fast screening *in vitro *method. Results show that glucose starvation combined with insulin stimulation greatly enhanced ^18^FDG PET signal from HUVEC and fibroblast monolayers. For optimum signal, we recommend a 2-hour starvation period in 3 mM glucose and 1% FBS containing DMEM, followed by a 1-hour ^18^FDG incubation (two hours did show more uptake, but the gain was not enough to compensate for the loss of signal due to ^18 ^F radioactive decay). These conditions improve the ^18^FDG PET signal without having detrimental effects on cell homeostasis and survival. Following such preconditioning treatment, a 15-30 minute stabilization period, where commercially available 5.5 mM culture medium containing 10% FBS is added to the incubation medium, is also advised to reduce ^18^FDG cell efflux. Using this method, detection thresholds of 36 ± 7 and 21 ± 4 cells were achievable for HUVEC and fibroblasts, respectively. Future plans include the validation of the optimized parameters in a more realistic 3D *in vitro *model. This study provides support to further develop and validate non-invasive and non-destructive imaging methods such as PET to monitor and characterize high cell density cultures in tissue engineering. Moreover, the data collected in this *in vitro *imaging experiment of vascular cells could be helpful in devising clinically relevant imaging protocols for studying various vascular diseases.

## Competing interests

The authors declare that they have no competing interests.

## Authors' contributions

JAC designed the protocols, performed experiments, analyzed data, produced figures, graphs and table, and drafted the manuscripts; JAR provided assistance to the protocol design and data analysis; JFB contributed to the scanning set up to perform PET and CT image acquisitions; PV contributed to the factorial design, data analysis and writing of the paper; RL contributed to the overall design of the experiment, data analysis and final edition of the paper. All authors have read and approved the final manuscript.
